# The Hippo-YAP/β-catenin signaling axis coordinates odontogenic differentiation in dental pulp stem cells: Implications for dentin-pulp regeneration

**DOI:** 10.1371/journal.pone.0326978

**Published:** 2025-06-26

**Authors:** Chang Chen, Qiqi Yun, Juanli Ran, Ziyao Zhou, Pengxiang Zhang, Rong Li

**Affiliations:** Department of Stomatology, The Second Xiangya Hospital, Central South University, Changsha, Hunan, China; Università degli Studi della Campania, ITALY

## Abstract

**Objective:**

This study investigated the interplay between Hippo-YAP and β-catenin signaling in regulating odontogenic differentiation of human dental pulp stem cells (DPSCs) and explored its potential implications for dentin-pulp regeneration.

**Methods:**

Using lentivirus-mediated YAP overexpression/silencing, β-catenin siRNA knockdown, and pharmacological Wnt inhibition (via WIF-1), we assessed DPSC proliferation, migration, mineralization, and molecular markers (via qRT-PCR, immunofluorescence). In vivo validation employed subcutaneous transplantation of DPSC-seeded scaffolds in immunocompromised mice.

**Results:**

YAP activation enhanced DPSC proliferation (1.44-fold), migration (1.39-fold), invasion (1.54-fold), and differentiation, as evidenced by elevated ALP activity (1.46-fold) and mineralization (1.36-fold). We observed transcriptional upregulation of odontogenic markers (RUNX2, DSPP, DMP1, OCN, ALP) and Wnt pathway components (β-catenin, Cyclin D1, c-Myc). Immunofluorescence revealed coordinated YAP and β-catenin expression patterns during differentiation. β-catenin silencing or Wnt inhibition abolished YAP-mediated functional enhancements and simultaneously suppressed YAP expression, partially confirming bidirectional regulation. *In vivo*, YAP-overexpressing DPSCs exhibited 1.27- to 1.62-fold induction of dentin-specific markers and β-catenin, whereas YAP silencing impaired these markers expression.

**Conclusions:**

Our findings demonstrate that coordinated YAP and β-catenin signaling drives DPSC odontogenesis, with potential implications for dentin regeneration. Although reciprocal regulation is evident, direct molecular interactions require further validation.

## Introduction

Tooth loss remains a major challenge in modern dentistry [1], driving the need for biologically driven regeneration strategies that recapitulate the structural and functional complexity of natural dentition [2]. Among stem cell sources, dental pulp stem cells (DPSCs) have emerged as a promising candidate due to their accessibility, multilineage potential, and robust reparative functions within inflammatory microenvironments [3]. Emerging paradigms recognize pulpitis not as an endpoint but as a dynamic niche where DPSCs activate odontoblast-like differentiation to mediate the balance between pathological and regeneration [4]. This process involves intricate coordination of signaling pathways, with the Wnt/β-catenin and Hippo-YAP signaling pathways standing out as evolutionarily conserved regulators of stem cell fate [5].

The Wnt/β-catenin pathway plays dual roles in DPSCs: maintaining homeostasis through the structural and adhesive functions of β-catenin, while directing odontogenic differentiation via transcriptional regulation [6]. Balanced Wnt activation enhances reparative dentinogenesis by synergistically promoting proliferation and differentiation. However, excessive signaling risks ectopic mineralization—a therapeutic challenge requiring precise control [7–9]. Complementing the Wnt/β-catenin pathway, the Hippo-YAP pathway has recently been implicated in mineralized tissue regeneration through its mechanosensitive regulation of stem cell plasticity [10]. The phosphorylation-dependent nucleocytoplasmic shuttling of YAP enables it to act as a molecular rheostat, enhancing osteogenic commitment while suppressing adipogenesis in mesenchymal stem cells [11]. Notably, La Noce et al. [12] demonstrated that hyaluronan-based matrices activate YAP/TAZ to drive osteogenic differentiation of DPSCs, highlighting the responsiveness of this pathway to microenvironmental cues. While these findings establish the importance of YAP in bone regeneration, its specific role in dentinogenesis remains underexplored—a critical gap given the distinct transcriptional programs governing odontoblast versus osteoblast differentiation.

Mounting evidence reveals cross-regulation between the Hippo-YAP and Wnt/β-catenin signaling pathways in tissue regeneration. YAP physically interacts with Wnt components (LRP6, DVL3) to amplify β-catenin signaling, while Wnt activation stabilizes nuclear YAP by inhibiting LATS1/2 kinase activity [13]. In bone regeneration, this crosstalk forms self-reinforcing loops that enhance osteogenesis, however, analogous mechanisms in dental pulp regeneration remain uncharacterized [14]. Crucially, the odontogenic program of DPSCs requires precise spatiotemporal control of matrix deposition—a process fundamentally distinct from osteoid formation [15]. Recent work shows that YAP/TAZ-mediated cell-matrix interactions are critical for stem cell fate determination, suggesting that microenvironmental stiffness or biochemical composition might modulate YAP/β-catenin crosstalk during dentinogenesis [16]. However, whether this axis operates as a master regulator of the odontogenic commitment of DPSCs—akin to its role in osteogenesis—remains a critical unanswered question.

This study investigated the potential involvement of YAP-β-catenin crosstalk in DPSC differentiation. Through systematic modulation of both pathways using lentiviral vectors (YAP overexpression/knockdown, β-catenin siRNA) and pharmacological inhibitors (WIF-1), we observed that YAP activation enhanced β-catenin-dependent odontogenic marker expression (RUNX2, DSPP, DMP1) and mineralization in vitro. In vivo transplantation experiments further demonstrated that YAP-overexpressing DPSCs generated more dentin-specific markers than controls. These data established YAP-β-catenin interaction as a critical regulator in tooth development, which highlights candidate pathways for future pulp regeneration strategies.

## Materials and methods

### Ethics statement

This study was approved by the Ethics Committees of Second Xiangya Hospital, Central South University, China. Human dental specimens were obtained with written donor consent. Animal experiments strictly adhered to institutional protocols approved by the Experimental Animal Ethics Committee of the Second Xiangya Hospital, Central South University, China.

### Culture of DPSCs

DPSCs were isolated aseptically from third molars of adult donors (18–25 years) between January-April 2022. Cells were cultured in α-Minimum Essential Medium (α-MEM) with 10% fetal bovine serum (FBS, v/v) and 1% penicillin-streptomycin (all reagents purchased from Gibco, Thermo Fisher Scientific, USA), maintained at 37°C/5% CO₂ with medium refresh every 2 days.

### Odontoblast differentiation of DPSCs

Log-phase cells were seeded into 6-well plates at 2 × 10⁵ cells/well. At 80% confluence, cultures were switched to odontogenic differentiation medium containing α-MEM with 10% FBS, 50 μg/ml ascorbic acid, 10 mM β-glycerophosphate, and 100 nM dexamethasone (Sigma-Aldrich, USA). Control groups received basal medium (α-MEM + 10% FBS). After 14 days of mineralization induction, cells were harvested for analysis. Differentiation progression was monitored using a phase-contrast microscope (Nikon, Japan).

### Cell transfection

Log-phase cells (1 × 10⁵ cells/well in 6-well plates) were divided into: (i) untreated controls; (ii) negative vector controls (empty lentivirus, Genechem Co., China); (iii) YAP-overexpression group (OE-YAP, LV-YAP lentivirus, Genechem Co., China); (iv) YAP-knockdown group (sh-YAP, LV-YAP-RNAi, Genechem Co., China); (v) combination group (LV-YAP1 + β-catenin siRNA, Ribo Life Science, China). Transfection used Lipofectamine 2000 (Thermo Fisher Scientific, USA; 5 μL siRNA + 5 μL reagent in 95 μL serum-free MEM). Viral transduction was enhanced with HitransG A/P enhancers (Genechem Co., China).

### Quantitative RT-PCR

Total RNA was extracted using TRIzol™ Reagent (Thermo Fisher Scientific, USA). cDNA synthesis utilized the Transcriptor First Strand cDNA Synthesis Kit (Roche Diagnostics, Switzerland). Quantitative PCR was performed with SYBR™ Green Master Mix (Roche Diagnostics, Switzerland). Gene expression levels were normalized to GAPDH and calculated via the 2^−ΔΔCt^ method. Primer sequences are provided in Table 1.

**Table 1:** Primer sequences.

Gene	Forward (5’ to 3’)	Reverse (5’ to 3’)
Runx2	CACAAGTGCGGTGCAAACTT	GCTTGCAGCCTTAAATGACTCT
OCN	CCTCACACTCCTCGCCCTAT	TGCTTGGACACAAAGGCTGC
DSPP	TAGCATGGGCCATTCCAGTTC	AGAGCCATTCCCTTCTCCCT
DMP1	TCTTTGTGAACTACGGAGGGTA	GGTCTTCATTTGCCAAGGGT
ALP	ATCTTCCTGGGCGATGGGAT	CACATATGGGAAGCGGTCCA
Yap	ATGAACTCGGCTTCAGGTCC	TGGTTCATGGCAAAACGAGG
β-catenin	GCTGGGACCTTGCATAACCT	TCCACTGGTGAACCAAGCAT
cyclin D1	GCTGCGAAGTGGAAACCATC	CCTCCTTCTGCACACATTTGAA
c-MYC	GTCAAGAGGCGAACACACAAC	TTGGACGGACAGGATGTATGC
GAPDH	CCATGGGTGGAATCATATTGGA	TCAACGGATTTGGTCGTATTGG

### Immunofluorescence

Following 14-day induction, cells in 12-well plates were fixed in 4% paraformaldehyde (pH 7.4, 25°C, 30 min), permeabilized using 0.3% Triton X-100 (30 min), and blocked with 5% BSA (1 h). Primary antibodies against YAP (1:50; 13584-1-AP, Proteintech, USA)) and β-catenin (1:50; 51067-2-AP, Proteintech, USA) were incubated overnight at 4°C, followed by fluorescent secondary antibody (1:200; SA00013-4, Proteintech, USA). Nuclei were counterstained with DAPI (WB017, Wellbio, China). Fluorescence images were acquired using a Motic BA210T microscope. Protein expression was quantified using Image J software by measuring the average optical density.

### Alkaline phosphatase (ALP) staining

Fixed cells were sequentially processed with ALP substrate (Sigma-Aldrich; 37°C, 12 h), cobalt nitrate (Aladdin; 37°C, 5 min), and ALP sulfide solution (Beyotime; 2 min). Mineralization was quantified through ImageJ (v1.54) integrated density analysis.

### Alizarin red staining

Fixed cells were stained with 2% Alizarin Red S (pH 4.2; Beyotime, China; 30 min, 50 rpm), water-destained, and imaged (Nikon bright-field microscopy). ImageJ (v1.54) quantified mineralization via integrated density analysis.

### CCK-8 assay

Cell viability was assayed with CCK-8 (Dojindo, Japan) following manufacturer’s protocol. DPSCs were plated in 96-well plates (5 × 10⁴ cells/well) and cultured overnight. After 24 h, cells were PBS-rinsed and exposed to 10% CCK-8 medium. Post 4-hour incubation, 450 nm absorbance was measured (Heales microplate reader). Triplicate data normalized to t = 0 controls determined proliferation rates.

### Scratch migration assay

Grid-marked 6-well plates were seeded with DPSCs (5 × 10⁵/well). At 90% confluence, monolayers were pipette tip-scratched perpendicular to grids, PBS-washed (3×), and cultured in serum-free medium. Wound closure was documented at 0/24/48 h using phase-contrast microscopy (Nikon). ImageJ (v1.54) quantified migration rates through wound healing (%) analysis (triplicate fields/well).

### Transwell

Transwell migration assays were performed with DPSCs (2 × 10⁶ cells in 100 μL serum-free medium) loaded into 8 μm chambers (Corning, USA). Lower chambers contained α-MEM/10% FBS. Following 48-hour culture under standard conditions, non-migrated cells were PBS-rinsed and swab-cleaned. Migrated cells were 4% PFA-fixed, crystal violet-stained, and counted using inverted microscopy (Nikon, Japan). Cell counts from three random fields/chamber determined migration capacity.

### Tooth slice/scaffold preparation

Ethically approved third molars (n = 30; donor age:15–25 years) from the Second Xiangya Hospital were processed under sterile conditions as follows: teeth were disinfected with 70% ethanol, horizontally sectioned using a low-speed dental handpiece with emery needle under PBS irrigation to prepare 1 mm-thick dentin slices (pulp chamber volume: 29–43 mm^3^). Scaffold fabrication included sequential steps: 1) NaCl filling (250–450 μm particles), 2) chloroform-dissolved PLLA infiltration (MW 250kDa, Boehringer Ingelheim, Germany), and 3) 24-h static salt leaching in distilled water. Constructs underwent graded ethanol hydrophilization (70–100%, 5 min/step) for enhanced wettability, followed by cryopreservation at −80°C in PBS. Each experimental group included ≥6 biological replicates.

### Cell seeding and transplantation of tooth slice in immunodeficient mice

Tooth slices were divided into four groups: (1) Matrigel (Corning, USA)-only control, (2) DPSCs/Matrigel, (3) YAP-overexpressing DPSCs (OE-YAP)/Matrigel, and (4) YAP-knockdown DPSCs (sh-YAP)/Matrigel. Bilateral implantation into 5–7-week-old male immunodeficient mice (n = 5/group, total 20 animals) was performed under pentobarbital sodium anesthesia (40 mg/kg intraperitoneal injection). Mice were maintained at 22 ± 1°C with 55 ± 10% humidity under a 12-h light/dark cycle. At 28 days post-implantation, euthanasia was conducted via intracardiac injection of pentobarbital sodium (150 mg/kg) followed by cervical dislocation, with death confirmed by absence of corneal reflex and sustained cardiac arrest (>5 min), consistent with AVMA guidelines. Explanted scaffolds were processed as described [17]: fixed in 4% paraformaldehyde (24 h), decalcified with 10% EDTA (pH 7.4, 14 days), and embedded in paraffin. Serial 6-μm sections were immunostained with anti- dentin sialophosphoprotein (DSPP; 1:200, Thermo Fisher Scientific, USA), anti- dentin matrix acidic phosphoprotein 1 (DMP1; 1:200, Thermo Fisher Scientific, USA), RUNX2 (1:200, Bioss, USA), β-catenin (1:200, PTG, USA), ALP (1:200, Abcam, UK) and anti- Osteocalcin (OCN; 1:200, PTG, USA) antibodies, visualized using DAB, and quantified via Image J (v1.54) by measuring average optical density across ≥3 sections per sample (n ≥ 3/group).

### Statistical analysis

Data are expressed as mean ± SD. Comparisons used Student’s t-test (two groups) or one-way ANOVA (multiple groups) in SPSS v17.0 (IBM, USA). Graphs were generated with GraphPad Prism 6.0. Significance was set at *P* < 0.05.

## Results

### YAP and β-catenin exhibit progressive upregulation during odontogenic differentiation

**Fig 1:**
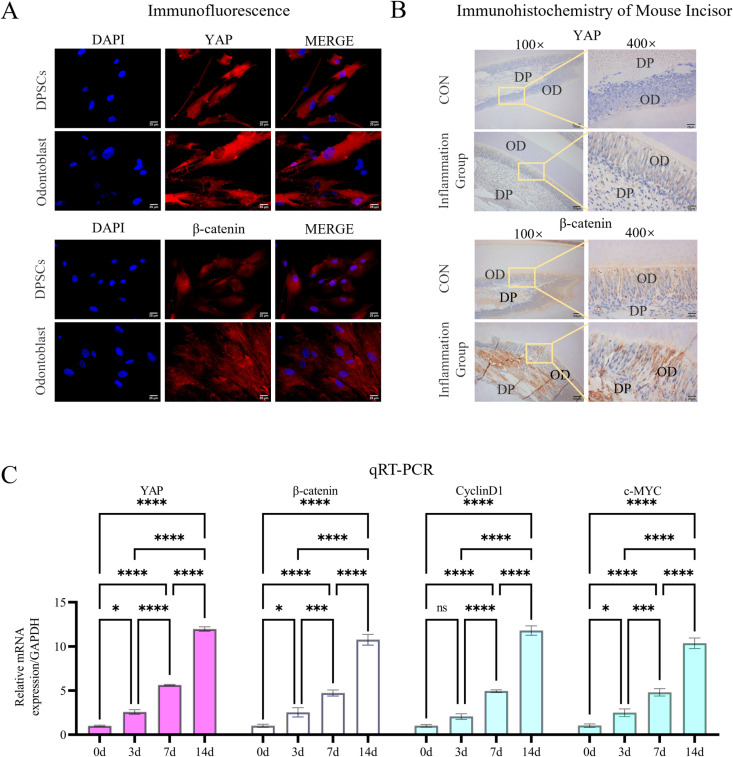
Spatiotemporal activation dynamics of YAP and β-catenin during DPSC odontogenic differentiation. **(A)** Immunofluorescence shows progressive increases in YAP and β-catenin protein levels in DPSCs over 14 days of odontogenic induction. Nuclei were stained with DAPI (blue). Scale bar: 25 µm. **(B)** Spatial immunohistochemical mapping in hydrogen peroxide-bleached murine incisors (mimicking inflammatory pulp conditions) demonstrates enriched YAP/β-catenin co-expression in odontoblast layers adjacent to predentin. Scale bar: 100 µm in100 × ; 25 µm in 400 × . (C) qRT-PCR quantification confirms progressive upregulation of YAP, β-catenin, and Wnt targets (Cyclin D1, c-Myc) during differentiation. Data: mean ± SD; *P < 0.05, ** *P* < 0.01, *** *P* < 0.001, **** *P* < 0.0001.

**Fig 2:**
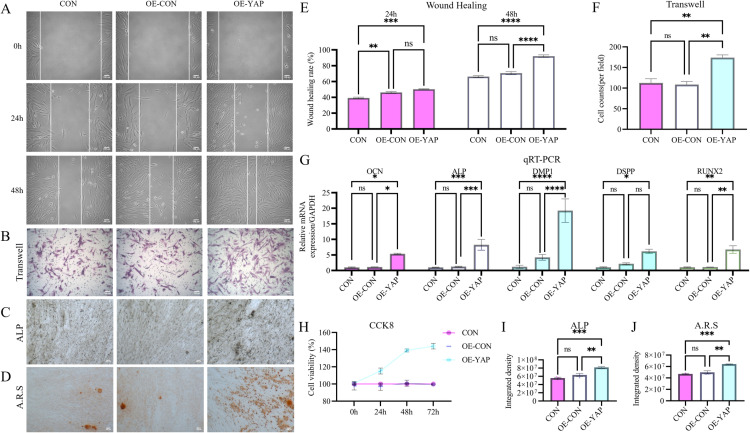
YAP overexpression potentiates DPSCs’ regenerative capacity. **(A, E)** Scratch wound healing assay shows a 1.39-fold accelerated closure in YAP-overexpressing DPSCs (OE-YAP) vs. controls at 48 h. **(B, F)** Transwell invasion assay demonstrates a 1.54-fold increased invasion capacity in OE-YAP cells. (C, **I)** Alkaline phosphatase (ALP) staining and activity quantification reveal a 1.46-fold elevation in OE-YAP cells at day 14. **(D, J)** Alizarin Red staining (red nodules) and mineralization quantification show a 1.36-fold higher mineralization in OE-YAP cells. (G) qRT-PCR confirms that OE-YAP upregulates odontogenic markers (RUNX2, DSPP, DMP1, OCN, and ALP). **(H)** CCK-8 proliferation assay shows a 1.44-fold increase in OE-YAP cells at 72 h. Data: mean ± SD; *P < 0.05, ** *P* < 0.01, *** *P* < 0.001, **** *P* < 0.0001. Scale bar: 100 µm.

To elucidate the functional involvement of YAP and β-catenin in odontogenic differentiation, we employed a multimodal analytical approach combining: (1) temporal immunofluorescence profiling of DPSCs under odontogenic induction (Fig 1A), and (2) spatial immunohistochemical mapping in post-bleached murine incisor sections (simulating inflammatory microenvironments; Fig 1B). Both proteins exhibited progressive upregulation during differentiation compared to pre-differentiation stages (S1A Fig). Notably, subcellular localization analysis revealed distinct spatial patterns: cultured DPSCs showed predominant cytoplasmic localization, whereas *in vivo* tissue sections displayed enrichment within the odontoblast layer adjacent to predentin. qRT-PCR further confirmed elevated mRNA levels of YAP and β-catenin during differentiation. Functional analyses demonstrated coordinated upregulation of Wnt targets Cyclin D1 (11.8 ± 0.91-fold) and c-Myc (10.36 ± 1.04-fold), which partially corroborated Wnt/β-catenin pathway activation (Fig 1C).

### YAP orchestrates DPSC proliferation, migration, invasion, and odontogenic differentiation

**Fig 3:**
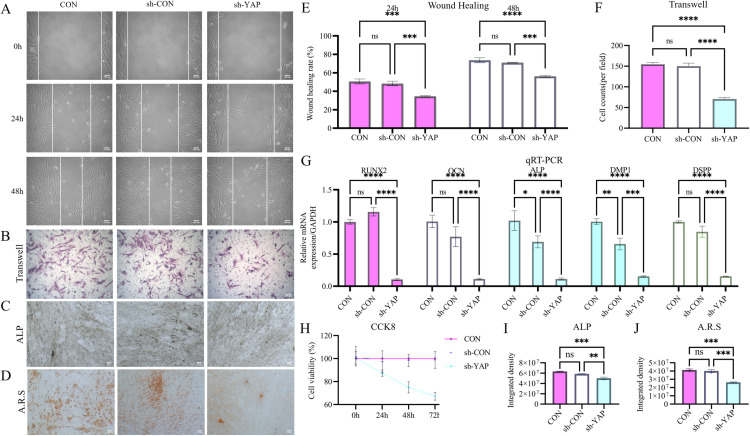
YAP knockdown suppresses DPSCs’ regenerative properties. **(A–J)** Functional assays demonstrate that YAP silencing (sh-YAP) significantly attenuates key regenerative capacities compared to controls: (i) wound healing **(A, E)**; (ii) Transwell invasion **(B, F)**; (iii) ALP activity (C, **I)**; (iv) mineralization **(D, J)**; (v) proliferation **(H)**. (G) qRT-PCR confirms that the sh-YAP group downregulates odontogenic markers. Data: mean ± SD; *P < 0.05, ** *P* < 0.01, *** *P* < 0.001, **** *P* < 0.0001. Scale bar: 100 µm.

**Fig 4:**
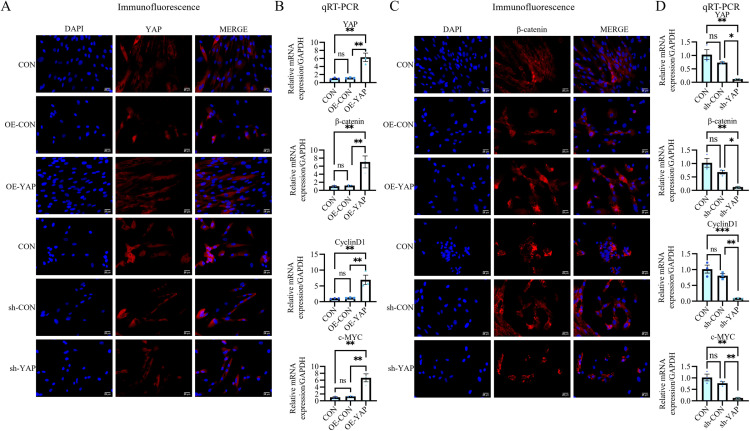
YAP enhances β-Catenin and Wnt-related gene expression in odontogenic DPSCs. **(A)** Lentiviral-mediated YAP overexpression (OE-YAP) and knockdown (sh-YAP) efficiency validated by immunofluorescence. (B, D) qRT-PCR shows OE-YAP upregulates Wnt pathway components (β-catenin, c-Myc, and Cyclin D1), while sh-YAP suppresses them. **(C)** Immunofluorescence reveals OE-YAP increases cytoplasmic β-catenin (red), whereas sh-YAP diminishes it. Data: mean ± SD; *P < 0.05, ** *P* < 0.01, *** *P* < 0.001, **** *P* < 0.0001. Scale bar: 25 µm.

Lentivirus-mediated YAP overexpression and shRNA knockdown in DPSCs (Fig 4A) revealed the functional role of YAP. YAP overexpression significantly enhanced proliferation (1.44-fold increase at 72 h; CCK-8 assay, Fig 2H), invasion (1.54-fold increase; Transwell assay, Fig 2B and 2F) and migration detected by wound healing assay (1.39-fold improvement; Fig 2A and 2E). Conversely, YAP knockdown attenuated these functional parameters by 24–54% compared to controls (Fig 3).

Odontogenic differentiation assays showed YAP overexpression-mediated transcriptional upregulation of key markers: RUNX2 (6.75 ± 2.23-fold), OCN (5.46 ± 1.21-fold), DSPP (6.57 ± 2.83-fold), DMP1 (19.41 ± 7.22-fold), and ALP (8.29 ± 3.03-fold) (Fig 2G). Functional assays corroborated these findings, showing 1.46-fold higher ALP activity (Fig 2C and 2I) and intensified Alizarin red-stained mineralization nodules at day 14 (Fig 2D and 2J). YAP knockdown suppressed marker expression by 85–89% and reduced mineralization to 36 ± 3% of control levels (Fig 3).

### YAP enhances β-catenin and Wnt signaling-related gene expression in odontogenic DPSCs

To investigate the mechanistic interplay between YAP and β-catenin signaling, we analyzed β-catenin expression under modulated YAP conditions. Immunofluorescence revealed that YAP overexpression significantly increased cytoplasmic β-catenin intensity (Fig 4A and S1B Fig), whereas YAP knockdown substantially reduced β-catenin levels in odontogenically differentiated DPSCs (Fig 4C and S1C Fig). Correlative upregulation of β-catenin (6.95 ± 2.17-fold), c-MYC (6.64 ± 1.79-fold), and Cyclin D1 (6.83 ± 2.1-fold) mRNA levels was observed upon YAP induction (Fig 4B). Conversely, YAP silencing drastically decreased their expression to 0.12-, 0.13-, and 0.10-fold of baseline levels, respectively (Fig 4D). These coordinated molecular changes establish YAP as a bifunctional modulator that enhances β-catenin activity and potentially orchestrates Wnt signaling activation.

### Bidirectional regulation of YAP/β-catenin coordinates odontogenic differentiation

**Fig 5:**
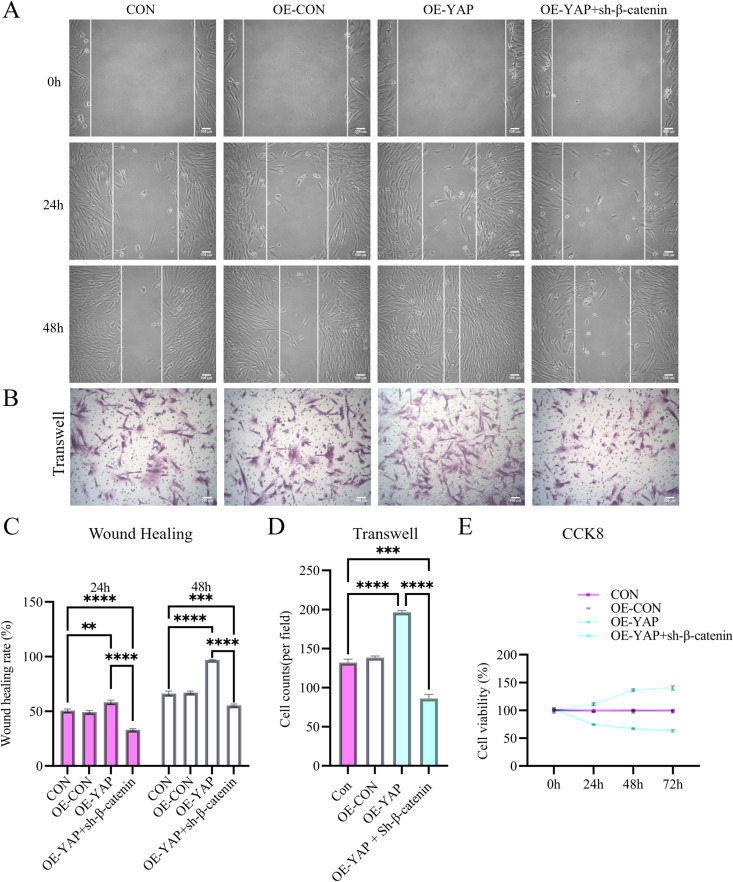
β-catenin ablation reverts YAP-driven functional enhancement. (A, C) The scratch wound closure assay: β-catenin siRNA reduces OE-YAP migration by 42.87% at 48 h (representative images in A, quantification in C). (B, D) The Transwell assay: β-catenin knockdown decreases OE-YAP invasion by 56.12% (representative images in B, quantification in D). (E) CCK-8 analysis shows that β-catenin siRNA reduces OE-YAP proliferative capacity by 54.97%. Data: mean ± SD; *P < 0.05, ** *P* < 0.01, *** *P* < 0.001, **** *P* < 0.0001 vs. control. Scale bar: 100 µm.

**Fig 6:**
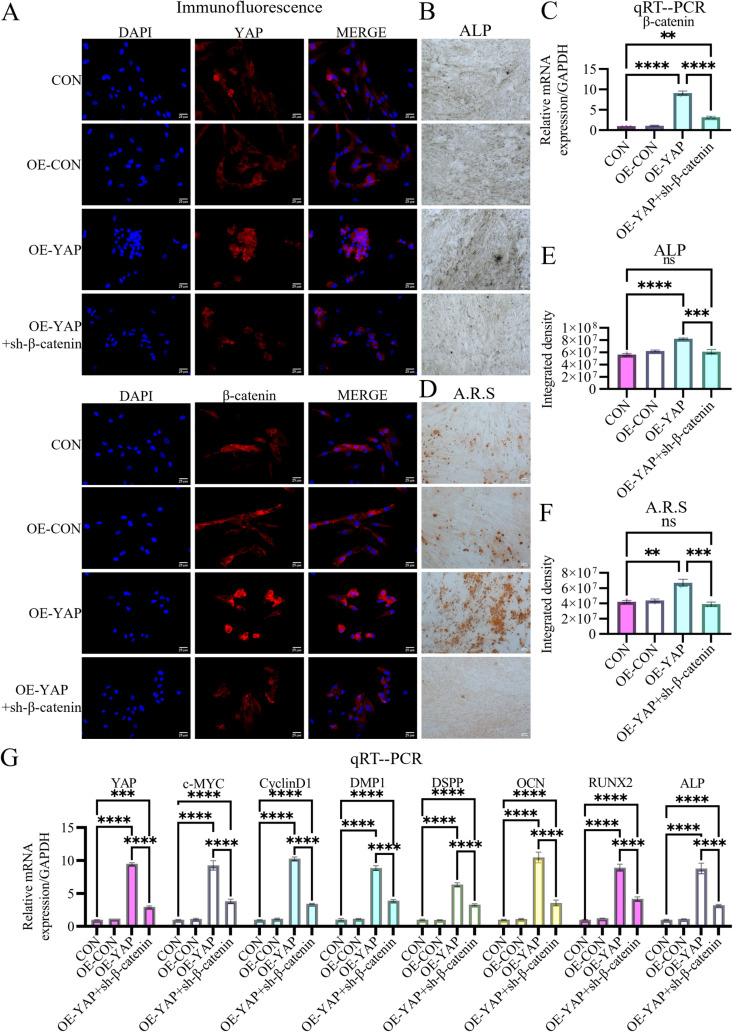
β-catenin ablation suppresses YAP expression and odontogenic capacity. (A) Immunofluorescence demonstrates that β-catenin knockdown not only abolishes OE-YAP-induced β-catenin upregulation but also reduces YAP expression, indicating β-catenin-dependent stabilization of YAP. Scale bar: 25 µm. (C) qRT-PCR validates β-catenin siRNA knockdown efficacy. (B, E) ALP activity is decreased by 25.48% in β-catenin-silenced OE-YAP cells compared to controls. Scale bar: 100 µm. (D, F) Mineralization capacity is reduced by 41.52% following β-catenin ablation. Scale bar: 100 µm. (G) β**-**catenin siRNA downregulates YAP-induced markers and reciprocally suppresses YAP mRNA expression. Data: mean ± SD; *P < 0.05, ** *P* < 0.01, *** *P* < 0.001, **** *P* < 0.0001.

To delineate the signaling hierarchy, we generated YAP-overexpressing DPSCs with simultaneous β-catenin siRNA knockdown (Fig 6A and 6C). Following 14-day odontogenic induction, β-catenin ablation counteracted YAP-driven functional enhancement, showing 54.97 ± 0.81% reduction in proliferation (CCK-8 assay; Fig 5E), 42.87 ± 1.88% delayed scratch wound closure (Fig 5A and 5C), and 56.12 ± 5.55% reduced Transwell invasion capacity (Fig 5B and 5D).

Molecular profiling revealed that β-catenin depletion significantly attenuated YAP-induced odontogenic marker expression, showing a 53.13 ± 3.88% reduction in RUNX2, 65.94 ± 6.19% in OCN, 48.09 ± 0.41% in DSPP, 55.67 ± 6.56% in DMP1, and 62.52 ± 7.52% in ALP levels (Fig 6G). Importantly, β-catenin silencing reciprocally suppressed YAP expression at both transcriptional (68.73 ± 3.93% mRNA reduction; Fig 6H) and translational levels (immunofluorescence-confirmed protein attenuation; Fig 6A and S1D Fig), partially revealing a bidirectional regulatory mechanism. This transcriptional repression correlated with diminished differentiation capacity, as evidenced by 25.48 ± 8.71% reduction in ALP activity (Fig 6B and 6E) and 41.52 ± 0.96% decrease in mineralization (Fig 6D and 6F).

**Fig 7:**
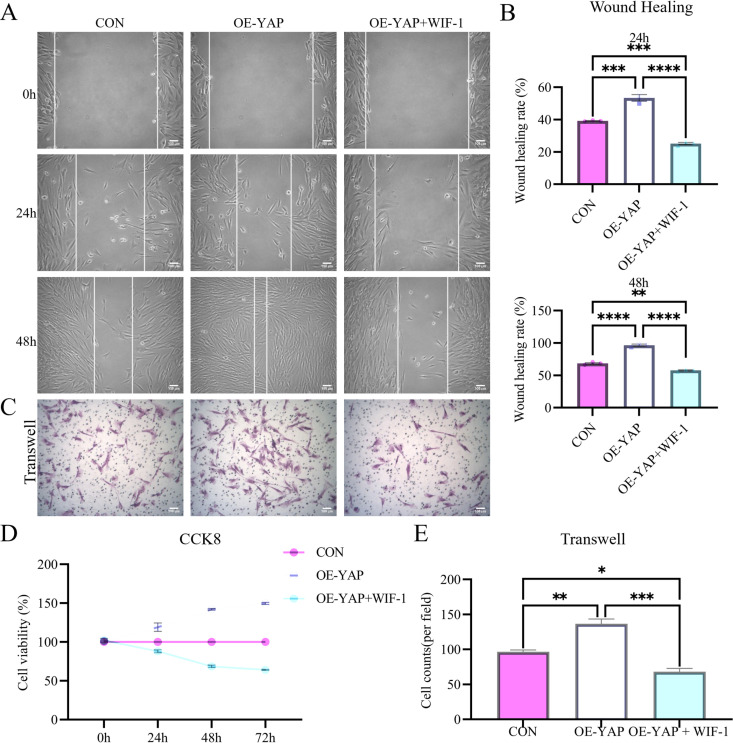
Pharmacological Wnt inhibition mimics β-catenin silencing in suppressing regenerative potential. **(A-D)** Wnt inhibitory factor-1 (WIF-1) treatment phenocopies β-catenin siRNA effects, including wound closure reduction (AB), invasion capacity decrease (CE), and proliferation decline **(D)**. Data: mean ± SD; *P < 0.05, ** *P* < 0.01, *** *P* < 0.001, **** *P* < 0.0001. Scale bar: 100 µm.

**Fig 8:**
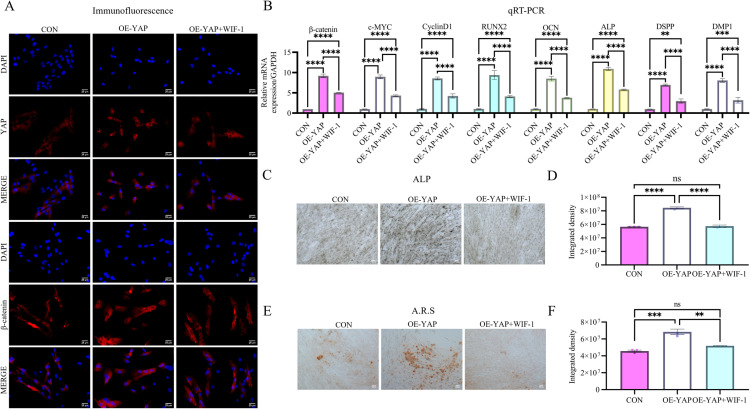
Pharmacological Wnt inhibition mimics β-catenin silencing in suppressing molecular expression and odontogenic differentiation. **(A)** Immunofluorescence demonstrates diminished expression of both YAP and β-catenin in WIF-1-treated OE-YAP cells. Scale bar: 25 µm (B) qRT-PCR confirms WIF-1-mediated downregulation of β-catenin and its downstream targets c-Myc and Cyclin D1 in OE-YAP cells. (CD) WIF-1 treatment abolishes YAP-mediated enhancement of ALP activity and mineralization capacity. Scale bar: 100 µm. Data: mean ± SD; *P < 0.05, ** *P* < 0.01, *** *P* < 0.001, **** *P* < 0.0001.

To confirm Wnt pathway involvement, we treated YAP-overexpressing DPSCs with the endogenous Wnt antagonist Wnt inhibitory factor-1 (WIF-1; 1 μg/mL for 48 h; R&D Systems, USA) as part of a dual-modulation strategy. Wnt pathway blockade, validated by reduced mRNA levels of β-catenin (44.75 ± 3.91%), c-MYC (51.64 ± 4.11%), and Cyclin D1 (49.96 ± 13.46%) (Fig 8B), phenocopied the functional and molecular effects of β-catenin ablation (Figs 7 and 8). This intervention induced comparable functional impairments and suppressed key molecular signatures, partially confirming the critical role of Wnt pathway activation in the YAP/β-catenin regulatory axis.

### *In vivo* validation of YAP/β-catenin axis in dentinogenesis

**Fig 9:**
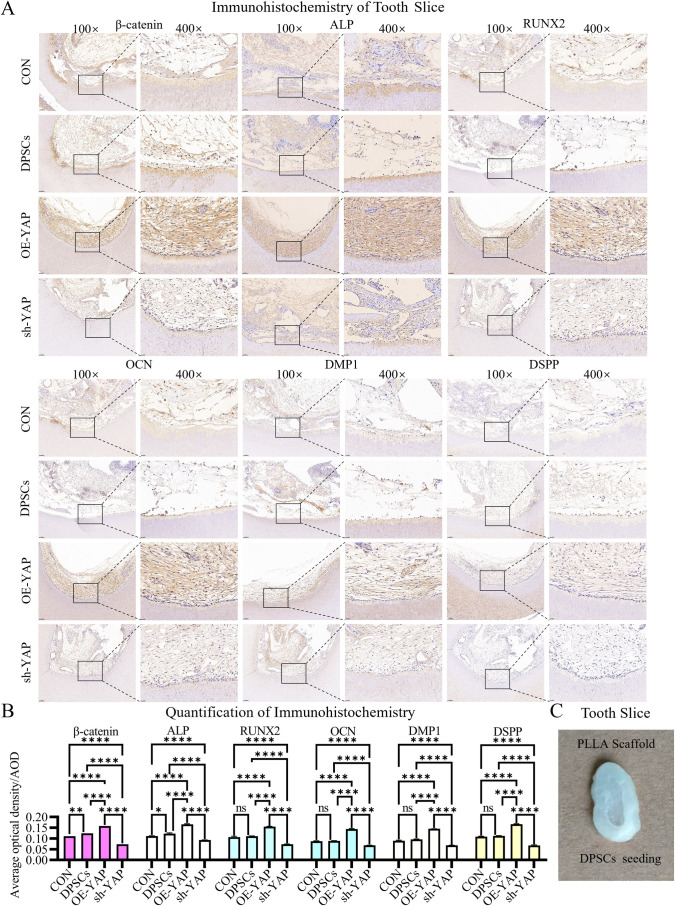
In vivo validation of YAP-mediated dentin regeneration. **(A)** Immunohistochemistry of implanted DPSCs grafts shows OE-YAP upregulates dentinogenic markers (DSPP, DMP1, RUNX2, OCN, and ALP) and β-catenin compared to controls. Scale bar: 100 µm (100×); 25 µm (400×). **(B)** Quantification confirms 1.36- to 1.62-fold induction of odontogenic markers and 1.27-fold β-catenin increase in OE-YAP grafts. **(C)** Schematic illustration of the tooth slice/scaffold design for ectopic transplantation. Data: mean ± SD; *P < 0.05, ** P < 0.01, *** P < 0.001, **** P < 0.0001.

For *in vivo* validation, tooth slices preloaded with odontogenically primed DPSCs (Fig 9C) were implanted into subcutaneous pockets of nude mice. Following 28-day implantation, immunohistochemical analysis (Fig 9A) revealed that YAP overexpression significantly increased expression of odontoblast-specific markers (RUNX2: 1.41-fold; OCN: 1.62-fold; DSPP: 1.49-fold; DMP1: 1.51-fold; ALP: 1.36-fold) compared to controls (Fig 9B). This was accompanied by a 1.27-fold increase in β-catenin expression (Fig 9B). Conversely, YAP silencing groups showed 50–70% reduction in marker expression compared to untreated controls. This *in vivo* evidence establishes the functional hierarchy of YAP-mediated odontogenic differentiation through β-catenin signaling.

## Discussion

This study elucidates a previously uncharacterized regulatory axis in DPSC biology, demonstrating that YAP coordinates odontogenic differentiation through bidirectional crosstalk with β-catenin signaling. While Hippo-YAP/β-catenin interactions have been implicated in osteogenesis [14], our findings reveal dentin-specific regulatory mechanisms, particularly the synergistic induction of DSPP and DMP1—key markers of odontoblast maturation that are absent in osteogenic lineages. This distinction underscores the necessity of tissue-specific pathway characterization, as dental pulp regeneration requires precise control of tubular dentin formation rather than osteoid matrix deposition [18]. Our work bridges a critical gap by demonstrating that YAP-mediated signaling converges with β-catenin activity to direct DPSCs toward a dentinogenic fate-a process mechanistically distinct from bone regeneration.

The scaffold microenvironment emerges as a potential modulator of this axis [19]. Our in vivo model utilized a PLLA/NaCl scaffold [20], functionally analogous to those of hyaluronan-based hydrogels described by La Noce et al [12], to provide synergistic topographical guidance and mechanical cues that orchestrate YAP/TAZ activation dynamics. Stiff substrates promote YAP nuclear translocation by enhancing cytoskeletal tension, whereas hydrogel elasticity regulates Wnt ligand bioavailability [21]. This interaction may explain the enhanced DSPP expression in transplanted YAP-overexpressing DPSCs, in which the scaffold’s 3D architecture likely amplified endogenous mechanosensitive signaling. Notably, the broader regenerative potential of DPSCs extends beyond dentin-pulp repair. Recent work demonstrates their exceptional capacity to integrate with biomaterial surfaces, which enhances implant osseointegration through ECM remodeling and paracrine signaling [22]. Future studies should delineate how scaffold stiffness or biochemical composition (e.g., hyaluronan incorporation) fine-tunes YAP/β-catenin dynamics to optimize dentin regeneration.

The highfold change in several markers may reflect both the potency of lentiviral YAP overexpression and the inherent responsiveness of DPSCs to YAP/β-catenin coactivation. While the magnitude of these changes exceeds physiological levels, it aligns with prior reports in DPSCs [23–25]. Nevertheless, transient activation strategies (e.g., scaffold-released YAP modulators) may better replicate physiological signaling dynamics in therapeutic applications.

Although our data support YAP/β-catenin crosstalk, the mechanistic underpinnings require further validation. First, while immunofluorescence and qRT-PCR analyses consistently indicate YAP/β-catenin coordination, Western blot quantification of protein-level interactions would strengthen these conclusions. Direct physical binding—as observed in osteoblastic systems [14]—remains to be conclusively demonstrated in DPSCs through co-immunoprecipitation or proximity ligation assays. Additionally, while subcutaneous implantation validates dentinogenic potential, orthotopic pulpitis models would better recapitulate the inflammatory and biomechanical milieu of clinical tooth repair. Future studies should also explore how scaffold properties (e.g., hyaluronan content) modulate YAP/TAZ-β-catenin crosstalk to optimize DPSC regenerative output.

By establishing YAP as a central regulator of dentinogenesis, this work identifies a therapeutic target for pulp regeneration. Small-molecule YAP activators (e.g., verteporfin analogs) or YAP-overexpressing DPSCs could enhance reparative dentin formation in vital pulp therapy. However, sustained Wnt activation risks pulp chamber obliteration, necessitating spatiotemporal control through biomaterial carriers such as injectable hydrogels with tunable Wnt/YAP release kinetics.

## Conclusions

Our data suggest potential crosstalk between the Hippo-YAP and β-catenin pathways in the odontogenic differentiation of DPSCs. YAP activation correlates with β-catenin upregulation, and their reciprocal modulation promotes mineralization. Further validation of direct interactions is required to clarify their therapeutic potential for dentin-pulp regeneration.

## Supporting information

S1 Fig Quantitative analysis of immunofluorescence. A Quantitative analysis of Fig 1A. B Quantitative analysis of Fig 4A and 4C (OE-YAP). C Quantitative analysis of Fig 4A and 4C (sh-YAP). D Quantitative analysis of Fig 6A. E Quantitative analysis of Fig 8A.
(TIF)
