# Supplementary figures and images for: The Hippo-YAP/β-catenin signaling axis coordinates odontogenic differentiation in dental pulp stem cells: Implications for dentin-pulp regeneration
Source: PLoS One. 2025 Jun 26;20(6):e0326978. doi: 10.1371/journal.pone.0326978 (PMC12200642; doi:10.1371/journal.pone.0326978)

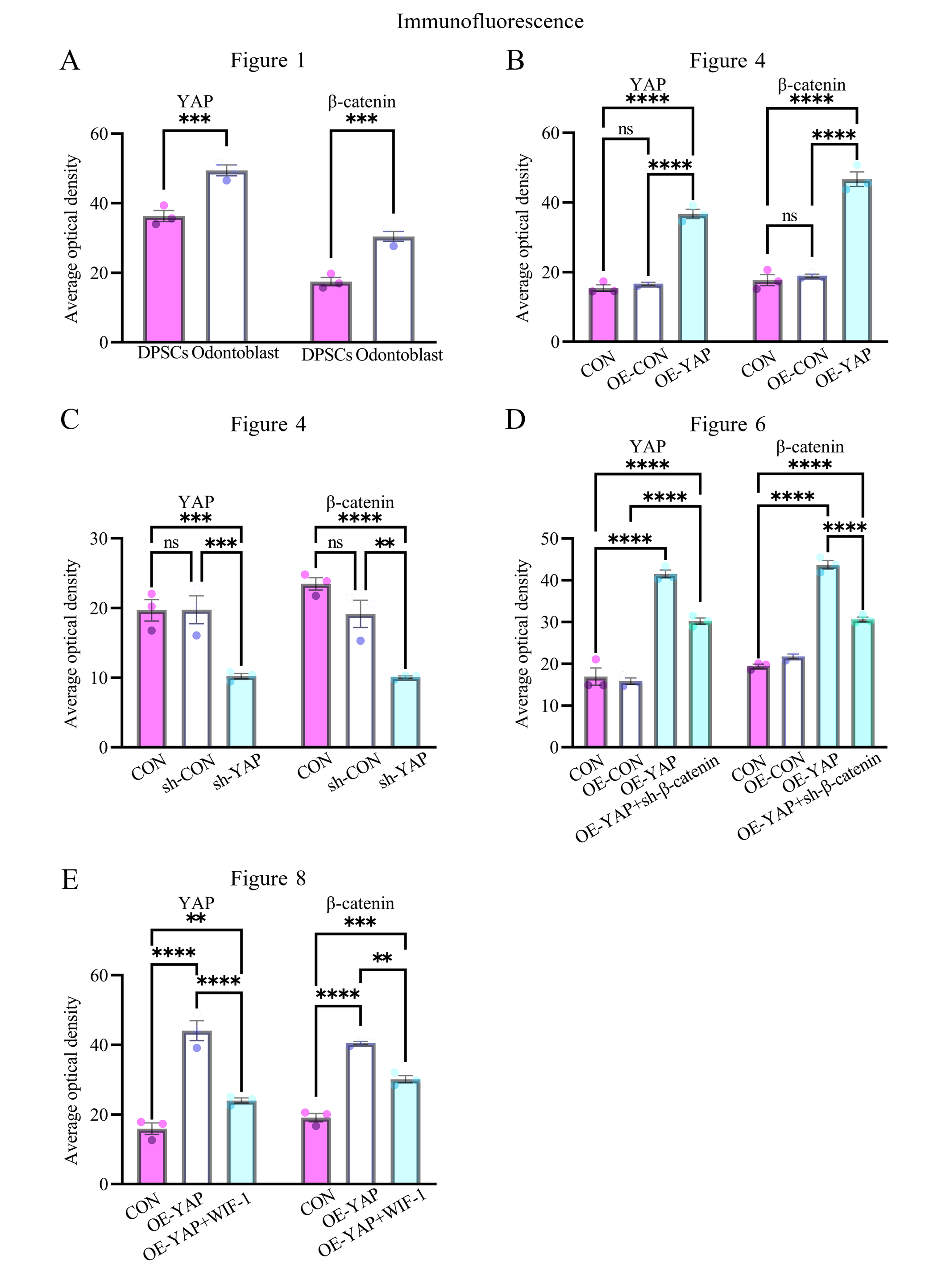

Supplement: S1 Fig — A Quantitative analysis of Fig 1A. B Quantitative analysis of Fig 4A and 4C (OE-YAP). C Quantitative analysis of Fig 4A and 4C (sh-YAP). D Quantitative analysis of Fig 6A. E Quantitative analysis of Fig 8A. (TIF) [file pone.0326978.s001.tif]
